# Long-lived excitons in thermally annealed hydrothermal ZnO

**DOI:** 10.1016/j.heliyon.2024.e26049

**Published:** 2024-02-08

**Authors:** Patrik Ščajev, Daniela Gogova

**Affiliations:** a Institute of Photonics and Nanotechnology, Faculty of Physics, Vilnius University, Saulėtekio Ave. 3, LT-10257, Vilnius, Lithuania; b Central Laboratory of Solar Energy and New Energy Sources at the Bulgarian Academy of Sciences, Tzarigradsko Chaussee Blvd. 72, 1784, Sofia, Bulgaria; c Department of Physics, Chemistry and Biology, Linkoping University, 583 30, Linkoping, Sweden

**Keywords:** ZnO, Excitons, Recombination, Time-resolved photoluminescence, Pump-probe

## Abstract

Applying thermal annealing to hydrothermal ZnO crystals an enhancement of exciton lifetime from 80 ps to 40 ns was achieved boosting PL quantum efficiency of the UV luminescence up to 70 %. The lifetime improvement is related to the reduced density of carrier traps by a few orders of magnitude as revealed by the reduction of the slow decay tail in pump probe decays coupled with weaker defects-related PL. The diffusion coefficient was determined to be 0.5 cm^2^/s, providing a large exciton diffusion length of 1.4 μm. The UV PL lifetime drop at the lowest exciton densities was explained by capture to traps. Release of holes from acceptor traps provided delayed exciton luminescence with ∼200 μs day time and 390 meV thermal activation energy. Pump-probe decays provided exciton absorption cross-section of 9 × 10^−18^ cm^2^ at 1550 nm wavelength and verified the PL decay times of excitons. Amplitudes and decay times of the microsecond slow decay tails have been correlated with the trap densities and their photoluminescence. A surface recombination velocity of 500 cm/s and the bimolecular free carrier recombination coefficient 0.7 × 10^−11^ cm^3^/s were calculated. Therefore, the properly annealed hydrothermally grown ZnO can be a viable and integral part of many functional devices as light-emitting diodes and lasers.

## 1 Introduction

ZnO is a wide bandgap semiconductor attracting considerable scientific and technological interest due to its unique properties such as a wide and direct bandgap [1], inexpensive Earth-abundant constituents relevant to the sustainable development [2], nontoxicity [3], recyclability, and last but not least the high exciton binding energy of 60 meV [4], superior in comparison to that of the other technology-important wide bandgap semiconductors like GaN [5], AlN [6], SiC [7]. The later advantage could lead to lasing action based on exciton recombination even above room temperature. ZnO has demonstrated a very large potential in various device applications such as photodetectors [8,9], photocatalysts [10,11], lasers [12], ZnO nano-based displays [13] and light-emitting diodes [14]. Hydrothermal synthesis of ZnO is of considerable interest due to its low cost, simplicity, and relatively low growth temperature (below 200 °C). Since the synthesis is performed in aqueous solutions, it is regarded as safe and environmentally friendly [15]. Moreover, ZnO is composed of Earth-abundant elements in contrast to group III nitrides [16].

Because of the relatively poor optoelectronic properties of the excitons in hydrothermally grown ZnO, the additional thermal annealing has been established as a process capable of improving the quality of the crystals by attaining longer lifetimes in the material due to the deep centers density reduction [17]. Oxygen annealing is shown to improve exciton photoluminescence in ZnO material [[18], [19], [20]]. An oxygen atmosphere prevents the oxygen loss from ZnO at high temperatures. Zn and O vacancy defects are annealed improving electrical quality, while annealing at too high temperatures can lead to the formation of additional oxygen-related defects [18]. Therefore, the annealing should be optimized in terms of temperature and duration. Particularly, thermal annealing in oxygen and vacuum were figured out to enhance the picosecond lifetimes up to the values of 2–6 ns [17]. In our previous research, we studied in detail a standard ZnO substrate, grown by hydrothermal method [21]. The exciton lifetimes measured were rather short - ∼220 ps, which are not application-relevant. Meanwhile, the defect emission was found to be much stronger than the excitonic one, revealing the need for their density reduction.

In the present work, we have achieved an improved excitonic lifetime by almost three orders of magnitude by oxygen annealing of the hydrothermal bulk ZnO. This allows gaining a deeper insight into the exciton transport dynamics and provides a technological approach for the development of effective optoelectronic applications based on a cost-efficient material. The obtained large diffusion length and high QE at large carrier densities gives a possibility for producing micrometer-thick hydrothermal ZnO light emitting devices which can have UV light output intensity by few orders of magnitude larger than standard thin-film LED technology. Recently, nanofin ZnO LED was developed with 1000 times larger light brightness than in available LED devices [22]. That was possible due to the absence of efficiency droop in ZnO, whereas droop is typical for commercial nitride LEDs at high carrier densities. Thereafter, hydrothermal ZnO UV LEDs could be applied for safe UVA disinfection of touch screens [23], pumping of white phosphors for lighting [24], and ultra-bright micro-LEDs for displays [25].

## 2 Samples and techniques

Bulk hydrothermally grown ZnO wafers (hexagonal structure: a = 3.252 Å, c = 5.313 Å), 2-inch in diameter and 500-μm-thick were purchased from MTI Corp. An as-received wafer was used as a reference sample and denoted as sample R. Two ZnO substrates, cut from a 2-inch wafer, were annealed at 950 °C and 1050 °C for 1 h in oxygen-containing atmosphere – named samples A1 and A2, respectively. The thermal annealing was carried out in a quartz tubular furnace under pure oxygen atmosphere. The pressure in the furnace was kept close to 1 atm. The temperature was ramped up from room temperature to annealing temperature with a rate of 25 K/min. The real annealing was maintained for 1 h. After the heating, the cooling down to room temperature appeared naturally. The substrate typical contamination, given in the manufacturer specification, in wt.% is Mg: <0.0005; Al: <0.0030; Si: 0.0030; Ti: 0.0010; Cu: <0.0030; Fe: <0.005 Ca: <0.0005; Ag: <0.0002; dislocation density <100 cm^−2^ [26].

Time-resolved photoluminescence (TRPL) measurements were performed in a standard back-scattering geometry using a Hamamatsu streak camera (C10627) attached to an Acton monochromator. For excitation, 180 fs pulses with a repetition rate of 10 kHz from an ORPHEUS parametric amplifier, pumped by a PHAROS laser, were used. Details can be found elsewhere [27]. The excitation pulses at 320 nm were used for one-photon (1P) surface excitation, while the 530 nm pulses were employed for the two-photon (2P) bulk excitation. The temperature-dependent measurements were performed in a nitrogen cryostat (CTI-CRYOGENICS).

The pump-probe (PP) setup is detailly described in Ref. [28]. Excitons/carriers (the term for both – electron–hole pairs) were excited by an above-gap optical pulse at λ_pump_ = 351 nm from a Q-switched Nd:YLF laser, emitting pulses with 12 ps duration. A single mode λ_probe_ = 1550 nm CW laser of 50 mW power (Eblana Photonics) was used for probing. For two-photon excitations λ_pump_ = 527 nm, 15 ps pulses were employed. Transmitted probe intensity variation with up to 100 ps time resolution was monitored using a 5 GHz bandwidth InGaAs biased photodetector (Thorlabs DET08CFC/M), a 4 GHz bandwidth low noise amplifier and a 6 GHz LeCroy oscilloscope (SDA 6000). Decay kinetics were averaged 50 times with and without excitation and transferred to the computer.

The ZnO internal quantum efficiency was measured using an integrating sphere (AvaSphere-150) with a waveguide spectrometer ASEQ Instruments LR1-T (200–1100 nm) at an excitation wavelength of 351 nm.

The diffusivity of electron–hole pairs was measured by LITG method using 12 ps Nd:YLF laser (PL2243, Ekspla) third harmonic (351 nm) for excitation, while 1053 nm probe pulses (1st harmonic) were delayed by a delay line (Aerotech ACT115DL); the method details can be found in Ref. [9]. The electron effective mass in ZnO is m_e_* = 0.22 m_0_ [29] and the hole effective mass is m_h_* = 0.6 m_0_ * [30] indicating that mainly electrons contribute to the DE signal as DE ∼ 1/m^2^.

## 3 Decay characterization

### 3.1 Photoluminescence characterization

**Fig. 1:**
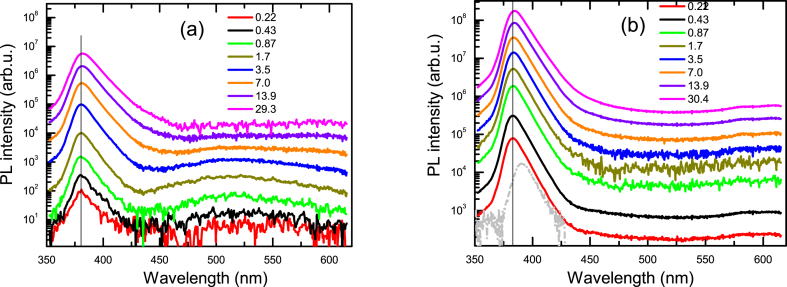
Excitation-dependent PL spectra in R (a) and A2 (b) samples at 320 nm excitation. The vertical line shows a 381 nm peak. Excitation fluences Φ are provided in μJ/cm^2^. In (b) dashed line shows the PL spectrum at 2P excitation.

**Fig. 2:**
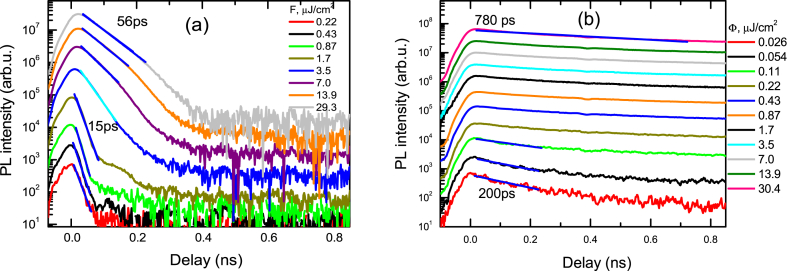
Excitation-dependent PL decays in R (a) and A2 (b) samples at 320 nm excitation.

The photoluminescence spectra of the samples R and A2 are depicted in Fig. 1a and b, respectively. After annealing the exciton peak position is not changed (381 nm), though the defects-related peak at 520 nm is significantly reduced. The defect emission can be related to shallow donor to deep acceptor O_Zn_, transitions [31]. The 520 nm green emission can also be associated to the shallow donor to V_O_ transitions [32]. Thus, the annealing process removes V_O_, remaining only a shallow acceptor, leading to a weak blue emission (440 nm). Moreover, the PL intensity of the excitons is much stronger in the annealed sample. This was explained by their much longer lifetimes as can be seen by faster PL decays in Fig. 2a in comparison to that in Fig. 2b. Fast initial parts in sample A2 can be attributed to the surface recombination and trapping at the lowest excitations. The PL peak shift to longer wavelengths by 10 nm due to reabsorption at 2P excitation (observed in Fig. 1b) reduces the light output by approximately 3 times as can be evaluated by integrating the PL spectra coinciding within the long wavelength range. Obviously, the bulk 2P PL emission extraction is 3 times lower and would not affect the PL decay time to the same extent as at surface excitation.

**Fig. 3:**
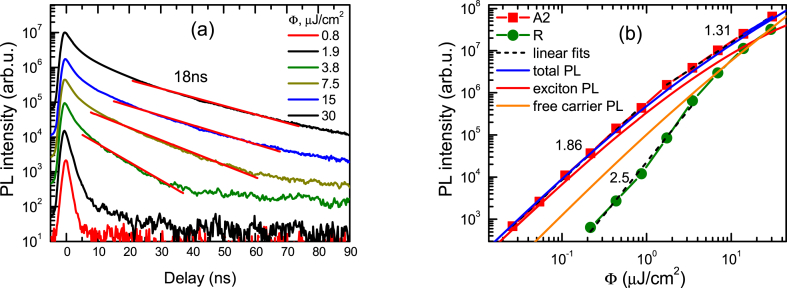
Slow PL decays in A2 sample (a) and excitation-dependent peak PL intensities (b). Excitation at 320 nm was applied.

The slow decay parts of sample A2 (illustrated in Fig. 3a) reach 18 ns long lifetimes confirming very high ZnO material quality. The low excitation decays are much faster due to trapping. The excitation-dependent peak PL intensity of excitons at low excitations has a slope of almost 2 in a log-log scale as depicted in Fig. 3b, however, at higher excitations, it reduces due to the exciton screening and emergence of a free carrier emission. The large initial slope shows an increase in the exciton density at low excitations.

**Fig. 4:**
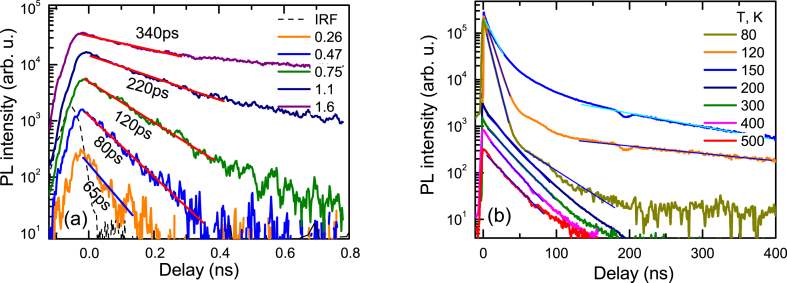
Two-photon excitation decay fast parts at 300 K (a) and temperature-dependent slow parts (b). Excitation fluences in (a) are provided in mJ/cm^2^. IRF shows instrumental response function. Solid lines are exponential fits.

**Fig. 5:**
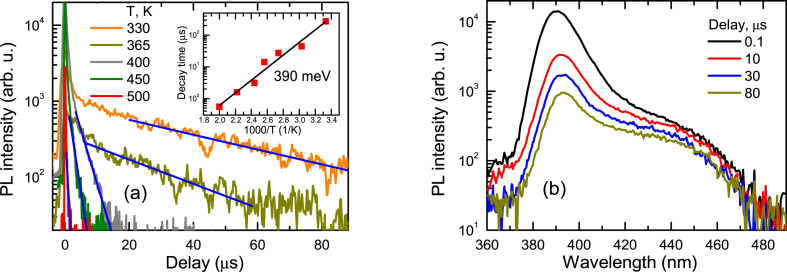
Delayed exciton luminescence decays vs. temperature (a) and time-dependent PL spectra at 300 K (b) at 2P excitation. In (a) the inset shows activation of the delayed decay time with temperature; solid lines are exponential fits.

The PL decays at 2P excitation are exhibited in Fig. 4a. The recombination time in this case increases with excitation indicating saturation of defects. The highest excitation leads to a 29.3 ns lifetime at 300 K (Fig. 4b). The temperature-dependent PL measurements show the variation of slow decays due to the increasing impact of radiative recombination rate at low temperatures for the initial part. At 120 K, and 150 K even slower decays in the tails are observed which can be attributed to the nonradiative carrier recombination as exciton density drops at large delays, and then excitons do not provide a significant radiative recombination contribution to the measured decay time. In Fig. 5 the slowest exciton emission decays are viewed.

In the delayed decays (see Fig. 5a) the acceptor traps provide holes by thermal activation, leading to the formation of excitons at very large delays, which can be called delayed luminescence. Excitons are formed when activated holes bind the electrons available from n-type doping of ∼10^15^ cm^−3^ (60 meV donors [21]), which can be related to Al impurities [33]. The slow decay activates with temperature with 390 meV energy. The additional slow PL peak, observed at 440 nm (Fig. 5b), indicates the trap emission (electron (or shallow donor) transition to shallow acceptor). This acceptor trap emission at 2.81 eV (Fig. 5b) provides ∼360 meV activation energy. That value is rather similar to the one determined from thermal activation indicating for the same acceptor as a hole trap. It can be a shallow acceptor V_Zn_ with around 300 meV activation energy [34]. This blue emission of the electron to shallow acceptor transition decays with a similar ∼170 μs day time as the delayed exciton emission, indicating that thermally activated holes recombine nonradiatively with the same fast exciton recombination time. The delayed exciton emission has a faster initial part (Fig. 5a) which can be explained by initially faster hole emission from the acceptors, as the hole emission rate is larger when most acceptors are in the neutral state [35].

### 3.2 Pump probe characterization

**Fig. 6:**
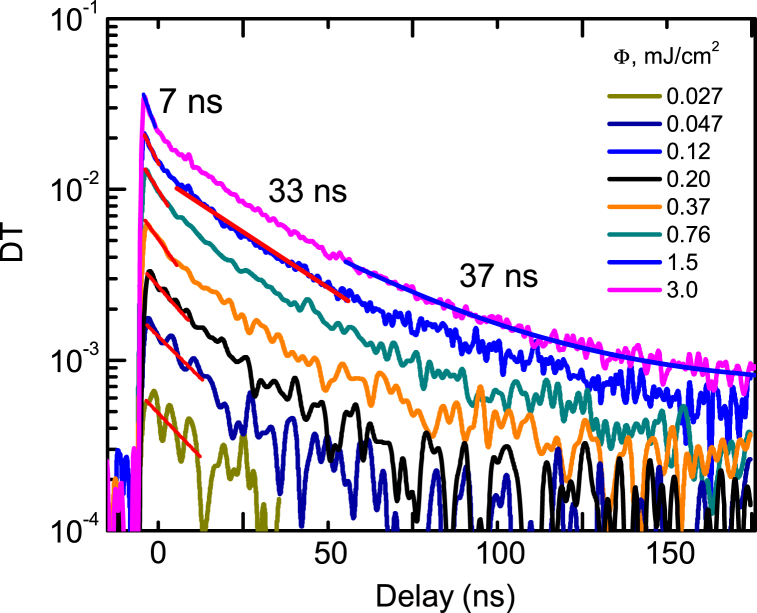
PP decays for the A2 sample at 1P excitation (351 nm).

**Fig. 7:**
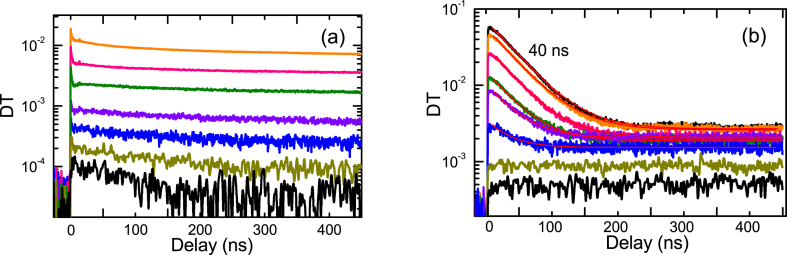
PP decays for R (a) and A2 (b) samples at 2P excitation. Curves in (b) are exponential fits with offsets.

**Fig. 8:**
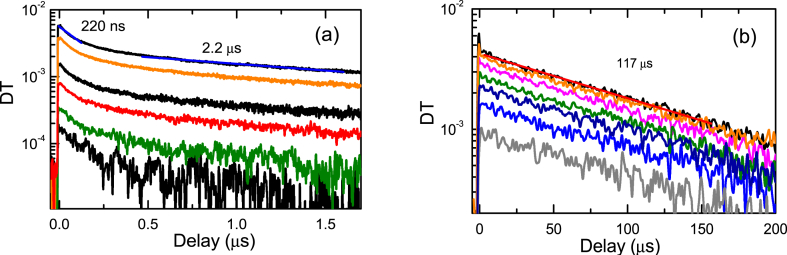
Slow PP decay parts at 2P excitation for the R (a) and A2 - (b) samples.

The excitation-dependent PP decays are revealed in Fig. 6 for the above bandgap excitation. The tail lifetime is similar to the one observed in PL decays. The fast part also appears at the highest excitations due to the nonlinear bimolecular recombination. Slower decays at 2P excitation in the R and A2 samples are compared in Fig. 7a and b. The R sample exhibits fast free exciton decay parts and a strong defect absorption tail. The annealed sample provides 40 ns exponential decay time which is limited dominantly by the nonradiative recombination, while the trap tail is much weaker. From Fig. 8 it is observed that the PP tail decay times are in the microsecond range and correlate with the slow defect PL emission illustrated in Fig. 5a.

The slow decays are more intensive and much faster in the R sample as revealed in Fig. 8a. This can be explained by the higher density of deep acceptors. The decay is nonlinear in the R sample indicating donor-acceptor pair recombination with different distances. In sample A2 the decays are almost exponential (Fig. 8b) due to the hole activation from shallow acceptor and further linear recombination with electrons by radiative or nonradiative mechanism. The signal from the slow traps in PP appears due to nonequilibrium free electrons. The decay time value of 117 μs is very similar to that of the 440 nm acceptor defect observed by PL.

**Fig. 9:**
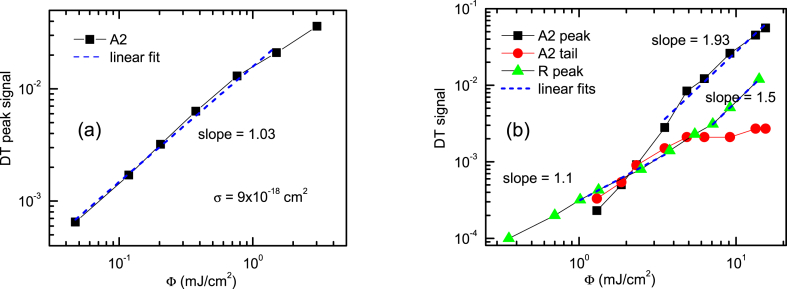
The PP signal excitation dependencies at 1P (a) and 2P (b) excitation (351 nm and 527 nm, respectively).

The 1P PP excitation dependence presented in Fig. 9a provides σ = 9 × 10^−18^ cm^2^ free exciton/carrier absorption cross section for the 1550 nm probe (equation DT = σΦ/hν was used [36]). The signal grows linearly with the excitation as the probe energy is much larger than the exciton binding energy, thus free exciton and free carrier absorption cross sections are similar [37]. In Fig. 9b comparison with the R sample at 2P excitation shows a linear defect absorption at low excitation in both samples. This indicates 527 nm sub-bandgap absorption between the ionized acceptor defect and the conduction band. In the sample A2 defects saturate, pointing to their density being lower. Free exciton/carrier absorption appears at medium excitations, and further increases quadratically for the A2 sample. This evidences a two-photon inter-band generation of the electron-hole pairs.

In comparison, a similar free electron absorption cross-section can be found in n-type ZnO at 1550 nm: σ_e_ = 10 × 10^−18^ cm^2^ [38], indicating PP signal is dominated by excess electrons (free or bound to shallow donors). Slow decays are also determined by nonequilibrium electrons when holes are bound to acceptors, thus electrons are not able to recombine. Release of holes from the acceptors leads to electron nonradiative recombination and delayed exciton luminescence as released holes bind to free electrons to form excitons.

## 4 Results and discussion

**Fig. 10:**
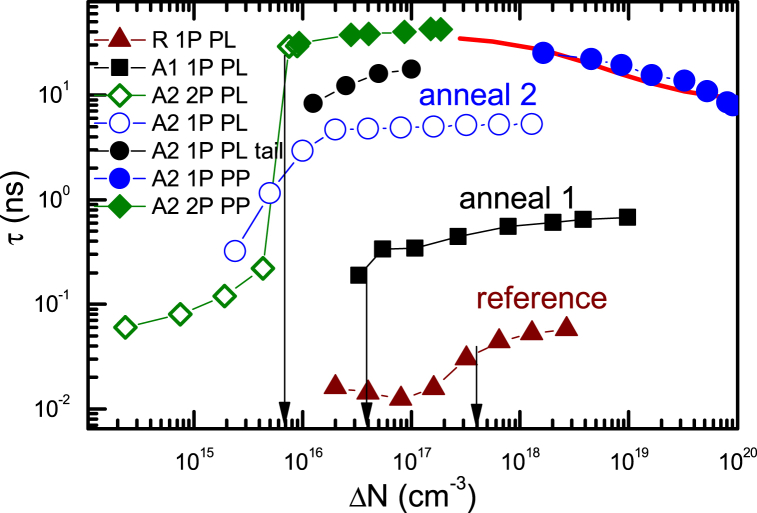
Excitation-dependent lifetime for the PL and PP decays in the samples studied. Vertical arrows exhibit the estimated trap densities. The solid line shows the nonlinear lifetime reduction fit including exciton, free carrier radiative and nonradiative recombination mechanisms at 1P excitation.

The decay time dependencies on excitation from the previous section are summarized in Fig. 10. Annealing shows strong lifetime improvement. The sharp lifetime drop at low excitations indicates the density of traps in the samples. Annealed samples show reduced trap densities to below 10^16^ cm^−3^. The PL and PP lifetimes well coincide. At the highest excitations, the PP lifetime decreases due to radiative recombination of free carriers. The ZnO absorption coefficient at 320 nm is very high α = 1.4 × 10^5^ cm^−1^ [39]. Therefore, the 1P PL decay times are faster in the initial part (about 5 ns) because of the exciton to depth diffusion leading to their density dilution and enhanced reabsorption [27], hence, their PL decay has a fast intensity reduction. The PP is free of this drawback as it integrates linearly the electron-hole pair signal over depth.

**Fig. 11:**
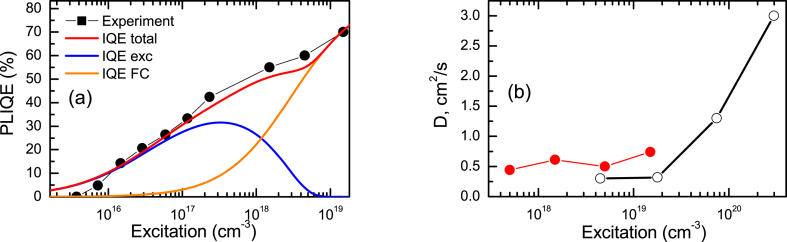
PL IQE (a) and diffusion coefficient (b) vs. electron-hole pair density at 351 nm excitation. In (a) solid lines indicate the calculated contributions of excitons (exc) and free carriers (FC) to the PL emission. In (b) open points show comparison data for hydrothermal ZnO studied in Ref. [21].

At later times after excitation, the excitons diffuse further from the surface reducing the impact of surface recombination. For the PP decay at t = 20 ns after excitation from Fig. 6 the decay time of τ_dec_ = 33 ns can be obtained. It differs from the bulk lifetime of τ_bulk_ = 40 ns (Fig. 7b). The surface lifetime in this case can be found from equation 1/τ_dec_ = 1/τ_bulk_ +1/τ_surf_. We find τ_surf_ = 189 ns. In this case, τ_surf_ = δ/S equation can be applied, where δ = (Dt)^1/2^ = 1.0 μm (for D see Fig. 11b). In this case a value of S = 530 cm/s can be calculated. With a similar approach the S = 450 cm/s can be obtained at 100 ns delay after excitation. The S value may be larger at a higher initial exciton density due to the effect of surface potential screening [40].

The exciton balance in ZnO is determined by their binding energy E_B0_ = 60 meV. The exciton density (ΔN_ex_) is described by the Sacha equations: ΔN_ex_ = ΔN_FC_^2^/n* with n* = N_dex_ exp(– E_ex_(ΔN_FC_)/k_B_T), ΔN = ΔN_FC_ + ΔN_ex_, E_ex_(ΔN) = E_B0_×(1–[ΔN_FC_/N_Mott_]^1/3^). Here ΔN_FC_ is the free carrier density, ΔN is the total excited electron-hole pair density, N_dex_ = 2(2πm_eh_k_B_T/h^2^) ^3/2^ = 1.6 × 10^18^ cm^−3^ is the exciton density of states, N_Mott_ is the Mott density [41]. The excited electron-hole pair density in the 1P case is ΔN = αΦ/(2hν), where α is the absorption coefficient 1.4 × 10^5^ cm^−1^ [39]. Here I is the excitation fluence, hν = 3.53 eV – excitation energy quanta at 351 nm. At two-photon excitations, the ΔN equations from Ref. [36] were used and the two-photon absorption coefficient β = 9 ± 1 cm/GW at 532 nm was determined.

The exciton oscillator strength decreases with the increase in the excitation intensity. This change is related to the decrease in the exciton binding energy in the highly excited state as E_ex_ ∼ E_B_^3/2^ [42], then exciton emission intensity or radiative emission rate R_ex_ = ΔN_ex_(E_b_(ΔN_FC_)/E_B0_)^3/2^/τ_radex_. Here τ_radex_ is the low density free exciton radiative lifetime. The free carrier radiative emission rate is R_FC_ = B_rad_(ΔN)ΔN_fc_^2^. Here the effective excitation-dependent bimolecular coefficient is B_rad_(ΔN) = B_0_/(1+ΔN_fc_/N_b_). Then, the total radiative emission rate is described by the equation R_rad_ = R_FC_ + R_ex_. The value of R_rad_ is proportional to the PL intensity. Exciton and free carrier contributions to the total PL intensity are shown in Fig. 3b. The bimolecular fit to the DT 1P initial lifetime in Fig. 6 is approximated as 1/τ(ΔN_fc_) = 1/τ_bulk_ + 1/τ_S_ + B_rad_(ΔN_fc_)ΔN_fc_, where B_0_ = (0.7 ± 0.1)x10^−11^ cm^3^/s is the nondegenerate bimolecular recombination coefficient, while the factor N_b_ = 1.3 × 10^19^ cm^−3^ appears due to carrier degeneracy (N_b_ limits the high injection free carrier radiative lifetime to 11 ns). A similar B_0_ value of 1.6 × 10^−11^ cm^3^/s was determined in ZnO [21]. For comparison, in GaN value of B_0_ = 2 × 10^−11^ cm^3^/s was observed [43].

The excitation dependence of the PL IQE, displayed in Fig. 11a, was fitted by means of the exciton balance equations and the 1/τ_dec_ = 1/τ_rad_ + 1/τ_nonrad_; IQE = τ_dec_R_rad_/ΔN relations. It is observed that excitons dominate the PL up to 3 × 10^18^ cm^−3^ excitation density. Later the free carrier bimolecular emission becomes dominant. The Mott density N_Mott_ = 2.5 × 10^18^ cm^−3^, and τ_radex_ = 10 ns were used in our calculations. The Mott density values in the literature vary within the 0.4-6x10^18^ cm^−3^ range [[44], [45], [46]]. ZnO quality and doping as well as experimental imperfections may affect the Mott transition density. The exciton radiative lifetime increases with temperature approximately as T ^−3/2^. Its value of 3 ns at 100 K determined in Ref. [47] allows estimating the RT value of 15 ns, which is similar to the obtained in the present work.

The determined diffusion coefficient (as a function of excitation) is illustrated in Fig. 11b. A value of approximately 0.5 cm^2^/s is observed. The comparison with the experimental data collected at higher excitation in Ref. [21] shows a sharp D increase due to the degeneracy of free carriers. The diffusion length L_D_ = (D × τ_bulk_) is calculated to be 1.4 μm in the A2 sample. The value is larger than in GaN crystals of different epitaxial technology, where dislocations have a strong impact on electrical properties [48].

**Fig. 12:**
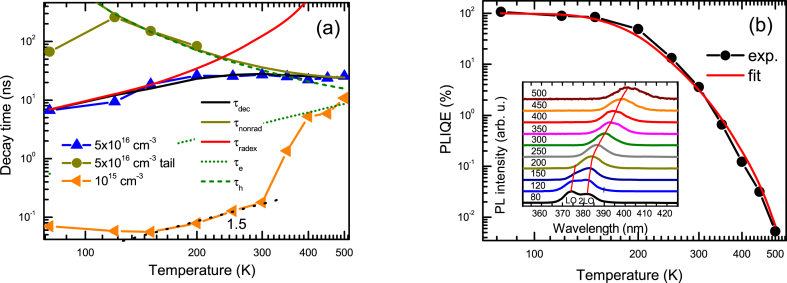
Temperature-dependent decay times at 5 × 10^16^ and 10^15^ cm^−3^ excited electron-hole pair densities in 2A sample (a); PL efficiency temperature dependence with a fit at 2 × 10^16^ cm^−3^ (b). In (b) inset shows temperature-dependent PL spectra at 2P excitation. Free exciton LO and 2LO peaks are indicated.

The temperature-dependent PL decay times at 2P excitations are provided in Fig. 12a for 2A sample. At low excitations (10^15^ cm^−3^) the PL decay times are fast (∼100 ps), which can be related to hole capture to V_Zn_^−^ acceptors. The electron and hole lifetimes are described by the relations [49]: τ_e_(T) = 1/(σ_e_(T)v_the_N_T_), τ_h_(T) = 1/(σ_h_(T)v_thh_N_T_). Here v_the_ and v_thh_ are the electron and hole thermal velocities (v_th_ = (8 kT/(m_e/h_π))^1/2^, m_e_ = 0.24 m_0_, m_h_ = 0.59 m_0_ for ZnO), with values 2.2 × 10^7^ cm/s, 1.4 × 10^7^ cm/s, respectively; N_T_ is the nonradiative trap density; σ_e_ and σ_h_ are the trap capture cross sections for electrons and holes, respectively. The charged V_Zn_^−^ acceptor density can be evaluated from Fig. 10 to be N_T_ = 8 × 10^15^ cm^−3^ (sharp lifetime increase at low 2P excitation). Therefore, for these acceptors σ_h_ = 9 × 10^−14^ cm^2^ at 300 K can be found. Such large capture cross sections of ∼10^−13^ cm^2^ are typical for shallow Coulomb traps [50]. With excitation increase the fast part becomes slower as the charged traps are neutralized by holes and bulk lifetime is observed at high excitation densities (thus we used 5 × 10^16^ cm^−3^ for the temperature measurements). At low temperatures capture to these Coulomb traps is faster and at the lowest ones becomes almost constant as the capture then becomes limited by multi-phonon emission [50]. On the other hand, at higher temperatures (>300 K) capture becomes slower due to stronger hole emission from the V_Zn_^−^ trap, and therefore its charged state density strongly reduces leading to an abrupt fast trapping lifetime increase (Fig. 12a), correlating with the delayed PL thermal activation (Fig. 5a).

The bipolar lifetime (when excited electron-hole density is above the trap and equilibrium carrier densities) in Fig. 12a shows a decrease at low temperatures. It is explained by reducing exciton radiative lifetime at low temperature (τ_radex_) and decreasing with temperature nonradiative lifetime (τ_nonrad_). The tail lifetime corresponds to the nonradiative process as described in Fig. 4b. The nonradiative lifetime at bipolar conditions (ΔN > n_0_) can be described as τ_nonrad_ (T) = τ_e_(T) + τ_h_(T) [35], where τ_e_(T) and τ_h_(T) are temperature-dependent electron and hole lifetimes described above. The nonradiative trap in ZnO can be attributed to oxygen vacancy (V_O_). Oxygen vacancies lead to green emission which is quenched after anneal in oxygen. It is known that the green PL intensity is proportional to the vacancy density [51]. The V_O_ trap density of ∼10^16^ cm^−3^ in untreated ZnO drops to ∼10^14^ cm^−3^ after annealling in an oxygen atmosphere at 1000 C for 1h [52].

The attributed oxygen vacancy is a double donor and electrons are captured to charged trap states V_O_^+^, thus capture cross section σ_e_(T) ∼ T^−2^ can be assumed (no barrier observed in the literature) [53]; for holes the capture to neutral trap V_O_^0^ can be assumed as σ_h_(T) ∼ T^0^exp(-E_b_/kT), with capture barrier to the trap E_b_ [53]. From the fit τ_nonrad_ (T) in Fig. 12a, the electron lifetime at 300 K was evaluated to be 4 ns. Using reference σ_e_ = 2 × 10^−13^ cm^2^ value [54], we obtain oxygen vacancy trap density V_O_ of 4 × 10^13^ cm^−3^, the latter value is similar to the literature values of 0.8-1x10^14^ cm^−3^ [51]. Before anneal the lifetime was ∼400 times shorter indicating a large V_O_ density of 1.6 × 10^16^ cm^−3^, and also explaining strong green emission. Similarly, in the literature, non-annealed ZnO showed densities ∼2 × 10^16^ cm^−3^ [52]. The hole capture cross section was fitted with E_b_ = 30 meV capture barrier. Hole capture cross section at 300 K was found 6 × 10^−14^ cm^2^, being smaller than that for electrons.

The temperature-dependent PLIQE at 2P excitation is provided in Fig. 12b. The PLIQE in the 80–150 K range is close to 100 %, due to fast radiative recombination and low reabsorption. At 300 K, the PLIQE at 1P excitation is about 3 times larger than at 2P excitation due to the reabsorption as evidenced in Fig. 1b. The ZnO exciton LO and 2LO peaks were identified at 80 K [55], LO peak disappears with temperature increase (see Fig. 12b inset). Reabsorption impact on PL emission collection is increasing rapidly above RT due to the increasing sub-bandgap absorption by LO phonons, explaining the excitonic LO peak disappearance effect [56]. The reabsorption can be described by the term c_reabs_(T) = 1/(1 +exp(α(T)d)), where d is the sample thickness, α(T) is the absorption coefficient at 2LO exciton emission peak. Then PLIQE = R_ex_(T)/(R_ex_(T) + R_nonr_ (T)) x c_reabs_(T), R_nonr_ (T) = ΔN/τ_nonrad_ (T). That PLIQE fit well describes the measured PLIQE dependence in Fig. 12b, indicating a strong impact of reabsorption to light extraction at elevated temperatures.

## 5 Conclusions

By applying thermal annealing to hydrothermally grown bulk ZnO we demonstrate enhanced exciton PL lifetime from 80 ps to 40 ns and suppressed green emission due to the reduced nonradiative V_O_ trap density below 10^14^ cm^−3^. Compensating V_Zn_ acceptor, leading to activated delayed exciton decays, density was estimated below 10^16^ cm^−3^. One- and two-photon excitations within pump-probe and photoluminescence decays allowed to discriminate the surface, bulk, nonradiative and radiative recombination coefficients. Modeling of the exciton balance has revealed that their emission is dominant up to the Mott transition and allows the achievement of 50 % IQE at room temperature. Temperature-dependent measurements reveal enhanced quantum efficiency at low temperatures due to shorter radiative and longer nonradiative lifetimes, and weaker reabsorption. Therefore, thermal annealing can serve as a perspective approach to improve the ZnO crystal's potential for diverse excitonic UV light emitting devices operating at room temperature.

## Data availability

The data that support the findings of this study are available from the corresponding author upon reasonable request.

## CRediT authorship contribution statement

**Patrik Ščajev:** Writing – review & editing, Writing – original draft, Visualization, Validation, Software, Resources, Project administration, Methodology, Investigation, Funding acquisition, Formal analysis, Data curation, Conceptualization. **Daniela Gogova:** Writing – review & editing, Validation, Resources, Methodology, Formal analysis, Conceptualization.

## Declaration of competing interest

The authors declare that they have no known competing financial interests or personal relationships that could have appeared to influence the work reported in this paper.
